# Is Metabolic Flexibility Altered in Multiple Sclerosis Patients?

**DOI:** 10.1371/journal.pone.0043675

**Published:** 2012-08-28

**Authors:** Anja Mähler, Jochen Steiniger, Markus Bock, Alexander U. Brandt, Verena Haas, Michael Boschmann, Friedemann Paul

**Affiliations:** 1 Experimental and Clinical Research Center, A Joint Cooperation between the Charité University Medicine Berlin and Max Delbrueck Center for Molecular Medicine, Berlin, Germany; 2 NeuroCure Clinical Research Center, Charité University Medicine, Berlin, Germany; Institut Pluridisciplinaire Hubert Curien, France

## Abstract

**Objectives:**

Metabolic flexibility is defined as ability to adjust fuel oxidation to fuel availability. Multiple sclerosis (MS) results in reduced muscle strength and exercise intolerance. We tested the hypothesis that altered metabolic flexibility contributes to exercise intolerance in MS patients.

**Methods:**

We studied 16 patients (all on glatiramer) and 16 matched healthy controls. Energy expenditure (EE), and carbohydrate (COX) and lipid oxidation (LOX) rates were determined by calorimetry, before and after an oral glucose load. We made measurements either at rest (canopy device) or during 40 min low-grade (0.5 W/kg) exercise (metabolic chamber). We also obtained plasma, and adipose tissue and skeletal muscle dialysate samples by microdialysis to study tissue-level metabolism under resting conditions.

**Results:**

At rest, fasting and postprandial plasma glucose, insulin, and free fatty acid levels did not differ between patients and controls. Fasting and postprandial COX was higher and LOX lower in patients. In adipose, fasting and postprandial dialysate glucose, lactate, and glycerol levels were higher in patients vs. controls. In muscle, fasting and postprandial dialysate metabolite levels did not differ significantly between the groups. During exercise, EE did not differ between the groups. However, COX increased sharply over 20 min in patients, without reaching a steady state, followed by an immediate decrease within the next 20 min and fell even below basal levels after exercise in patients, compared to controls.

**Conclusions:**

Glucose tolerance is not impaired in MS patients. At rest, there is no indication for metabolic inflexibility or mitochondrial dysfunction in skeletal muscle. The increased adipose tissue lipolytic activity might result from glatiramer treatment. Autonomic dysfunction might cause dysregulation of postprandial thermogenesis at rest and lipid mobilization during exercise.

## Introduction

Multiple sclerosis (MS) is the most common non-traumatic autoimmune neurological disease among young adults. MS is characterized by focal lymphocytic infiltration leading to demyelination and neuroaxonal loss. [1] Irreversible neuroaxonal damage results in a wide range of symptoms, including complex functional impairments such as abnormal walking mechanics, poor balance, muscle weakness, and exercise intolerance. [2] Altered mitochondrial function may contribute to the pathogenesis of axonal damage in MS. However, the data are primarily derived from studies on animal models or *post mortem* investigations in MS patients. In experimental autoimmune encephalomyelitis (EAE), mitochondrial dysfunction plays a pivotal role in axonal degeneration. [3] Recently, Nikić et al. suggested that demyelination may not be a prerequisite for axon damage, as commonly believed. Instead, they suggested focal intra-axonal mitochondrial pathology precedes changes in axon morphology in EAE. [4] Reduced respiratory chain complex IV activity has been observed within demyelinated axons in MS patients. [5], [6] In Huntingtońs disease, faulty complex I activity, as well as other deficits in muscle mitochondrial oxidative metabolism have been observed. [7], [8] Furthermore, investigators recorded increased energy expenditure during both fasting and hyperinsulinemic-euglycemic conditions. [9] Interestingly, extra-mitochondrial glucose metabolism in the CNS is increased in MS and is associated with disease progression. [10] A recent study reported significantly reduced complex I activity in freshly isolated mitochondria from skeletal muscle in Persian MS patients. [11].

Central and peripheral mitochondrial dysfunction should affect the regulation of systemic and local energy metabolism at rest, postprandially or during exercise. Mitochondrial dysfunction might also impair metabolic flexibility, i.e. the capacity to utilize lipid and carbohydrate fuels and to transition between the two oxidative fuel sources and therefore the ability to adjust fuel oxidation to fuel availability. [12] Therefore, we tested the hypothesis that metabolic flexibility is reduced in MS patients. We reasoned that a reduced metabolic flexibility might contribute to exercise intolerance above-and-beyond neural dysfunction. Impaired oxidative metabolism due to altered mitochondrial function results in increased lactate production and an elevated lactate/pyruvate ratio. [13], [14] Alternatively, an impaired function of the autonomic nervous system might also impair substrate mobilization and hence contribute to exercise intolerance.

Metabolic flexibility is mostly assessed by testing the switch from fat to carbohydrate oxidation 1) within an euglycemic hyperinsulinemic clamp or during overnight fasting (short-term), or 2) during a 3–7 d high-carbohydrate or high-fat diet (long-term), both combined with calorimetric measurements in order to assess changes in carbohydrate and lipid oxidation rates. [15] We relied on a standardized protocol that we designed to study metabolic alterations in patients with lipodystrophy. [16], [17].

## Methods

### Subjects

**Figure 1:**
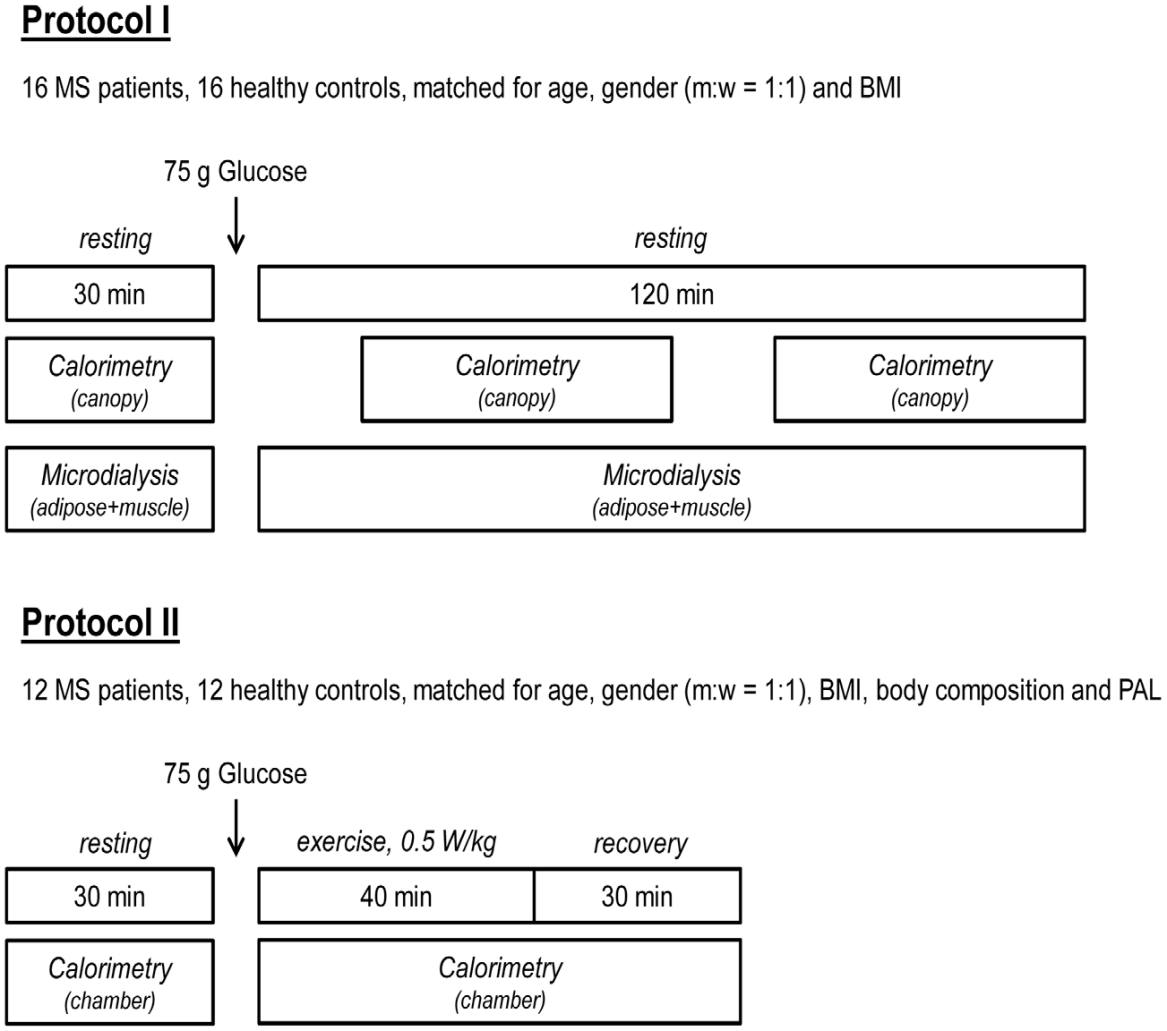
Study design. PAL, physical activity level; BMI, body mass index.

We recruited 16 consecutive patients with relapsing-remitting MS according to the 2005 panel criteria. [18] We also identified 16 healthy control subjects closely matched for gender, age, and body mass index (Fig. 1). The two groups did not differ significantly regarding demographic and anthropometric characteristics (Table 1). The institutional review board of Charité University Medicine Berlin approved the study and written informed consent was obtained from all participants prior study entry. Key inclusion criteria for MS patients were a stable immune-modulating, neuroprotective therapy with glatiramer for at least 12 months and an Extended Disability Status Scale (EDSS) score <5 but no other medication. [19].

**Table 1:** Demographic and anthropomorphic characteristics of MS patients and control subjects^a^.

Characteristics	Protocol I		Protocol II	
	MS	CTRL	MS	CTRL
Men/women (n)	8/8	8/8	6/6	6/6
Age (yrs)	45±6 (35–61)	40±12 (26–61)	46±7 (35–62)	48±9 (29–59)
BMI (kg/m^2^)	25.2±5.2 (19.1–37.7)	23.2±2.4 (19.3–29.3)	24.4±4.7 (19.5–36.3)	23.4±1.8 (19.3–28.0)
Body fat (%)	n/a	n/a	31±12 (16–50)	26±12 (6–39)
Lean body mass (%)	n/a	n/a	69±12 (50–84)	74±12 (61–94)
PAL	n/a	n/a	1.79±0.33 (1.37–2.42)	1.79±0.15 (1.56–2.16)
Disease duration (yrs)	11.1 (2.4–19.2)^b^	n/a	11.8 (3.5–19.9)^b^	n/a
EDSS (arbitrary units)	2.0 (1.0–4.5)^b^	n/a	2.0 (1.0–4.5)^b^	n/a

a Data are given as means ± SD (total range) unless stated otherwise. BMI denotes body mass index, WHR waist-to-hip ratio, PAL physical activity level, EDSS extended disability status scale, and n/a not applicable.

b Median (total range).

### Protocol

After a 12 h overnight fast, a catheter was placed in a large antecubital vein for blood sampling. One microdialysis probe (CMA/60, Dipylon Medical AB, Solna, Sweden) each was inserted into abdominal subcutaneous adipose tissue and *Vastus lateralis* muscle. [16], [17] After probe insertion, tissue perfusion was started with lactate free Ringer’s solution supplemented with 50 mmol/L ethanol at a flow rate of 2 µL/min using CMA/102 microdialysis pumps. Ethanol was added to assess changes in tissue perfusion by using the ethanol dilution technique. [20], [21] After a recovery period of at least 60 min, a 75 g oral glucose load (OGL) was given (300 ml solution, Dextro®, O.G.T., Hoffmann-La Roche AG, Grenzach-Wyhlen, Germany). Blood and dialysate samples were taken at baseline and every 15 min over 2 h after glucose. Carbon dioxide (CO2) production and oxygen (O2) consumption were measured by a canopy calorimeter (DeltatracII, Datex Ohmeda, Duisburg, Germany) in the supine position to calculate resting and postprandial energy expenditure (EE) and respiratory quotient (RQ  =  VCO2/VO2). The RQ can be used to assess changes in carbohydrate and lipid oxidation rates. Energy expenditure and substrate oxidation rates were calculated according the equations proposed by Ferrannini. [22].

Twelve MS patients and 12 healthy controls, closely matched for gender, age and body weight were able to perform standardized exercise in a metabolic chamber (Fig. 1). To further minimize a possible bias between the groups, patients and healthy controls were additionally matched for body composition (fat mass and fat-free mass) and physical activity level. Body composition was determined by measuring body weight and body volume by using an Air-Displacement Plethysmograph (Bod Pod, Life Measurements, Inc., Concord, CA). All participants completed a questionnaire asking for work, leisure, and sports activities within the preceding year. [23] Then, resting EE per hour was predicted according to the equations by Müller et al. [24] for normal weight subjects and the respective values were multiplied by energy cost and duration (h/wk) of the respective activity (Table 1). [25].

The metabolic chamber is a comfortable, airtight room (width: 2.5 m, depth: 2.0 m, height: 2.2 m) that is constantly supplied with fresh air like an open circuit indirect calorimeter. Measurements and calculations were the same as for the canopy calorimeter. In previous studies on 16 healthy subjects, the maximum spontaneous change in metabolic rate over a 3 h period was 0.2±0.09 kJ/min (3%). While being seated in a comfortable chair, measurements were started at first for air equilibration (30 min) and then for calculating subjects resting EE (40 min). Then, a 75-g oral glucose load was given. After 15 min, subjects started exercising over 40 min on a bicycle ergometer (VIAsprint 150 P, Ergoline, Bitz, Germany) adjusted individually to a workload of 0.5 W/kg body weight.

### Assays

Plasma glucose and insulin concentrations were measured according to international standards; free fatty acids (FFA) by an automated colorimetric test (ABX Pentra 400 Chemistry Analyser, Horiba ABX, Bedfordshire, U.K.).

Perfusate (inflow) and dialysate (outflow) ethanol concentrations were measured with a standard spectrophotometric enzymatic assay. A decrease in the ethanol outflow/inflow ratio (“ethanol ratio”) is equivalent to an increase in blood flow and vice versa. [20] Dialysate glucose, lactate, pyruvate, and glycerol concentrations were measured with the CMA/600 analyzer. In situ dialysate recovery for these metabolites was about 30% in adipose tissue and 50% in skeletal muscle, respectively, as assessed by near-equilibrium dialysis. [26].

### Statistics

All data was tested for normal distribution using histograms, Shapiro-Wilk tests and analysis of skewness and kurtosis. Since not all data were normally distributed, non-parametric tests were applied in all cases. Owing to this approach, it was not possible to correct for possible confounders as covariates. Consequently, patients and controls were closely matched regarding age and gender. For protocol I, group differences were analysed using Brunner’s non-parametric analysis for longitudinal data. [27] As results, group differences and group*time interaction effects from the ANOVA type model for small sample sizes are given. For protocol II, group differences were analyzed using Mann-Whitney-U tests for baseline (0 min) RQ and exercise EE (40 min). Brunner analysis was performed using R Project 2.14.1 with the macro F1_LD_F1. All other statistical analyses and graphics were performed with GraphPad Prism (version 5.01). All data in graphics are shown with mean and standard error of the mean (SEM). A *P* value <0.05 indicated statistical significance. All tests should be understood as constituting exploratory data analysis. Therefore, no previous power calculation or adjustments for multiple testing were made.

## Results

**Figure 2:**
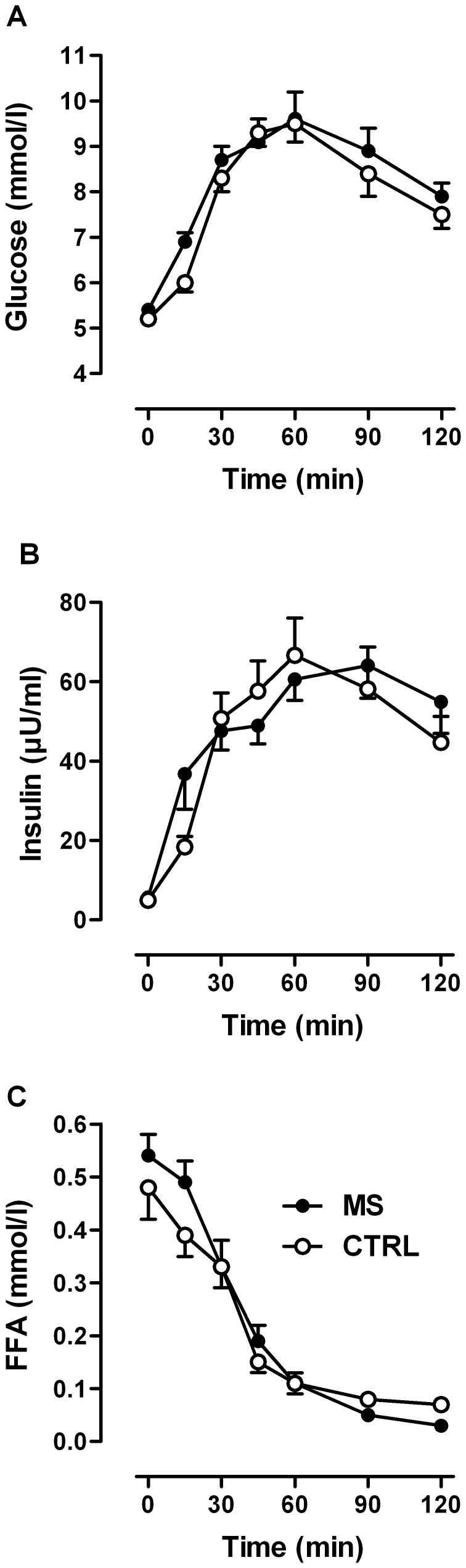
Systemic responses to oral glucose load. Serum (A) glucose, (B) insulin, and (C) free fatty acids (FFA) in MS patients (n = 16) and in control subjects (n = 16) before and after an oral glucose load. Data are given as means ± SEM. (Conversion factors to convert to Metric Units (mg/dL) are as follows: 18.02 for glucose, 0.028 for FFA).

Baseline values and postprandial profiles of plasma glucose, insulin, and FFA concentrations did not differ between the groups (Fig. 2). Fasting *ß*-hydroxybutyrate concentrations were 133 (SD, 174) *µ*mol/L in patients and 74 (SD, 57) *µ*mol/L in controls (not significant). Triacylglycerol concentrations did not differ between the groups, either before (controls: 101 (SD, 45) mg/dL, patients: 106 (SD, 46) mg/dL) or 2 h after glucose (controls: 89 (SD, 42) mg/dL, patients: 91 (SD, 49) mg/dL).

**Figure 3:**
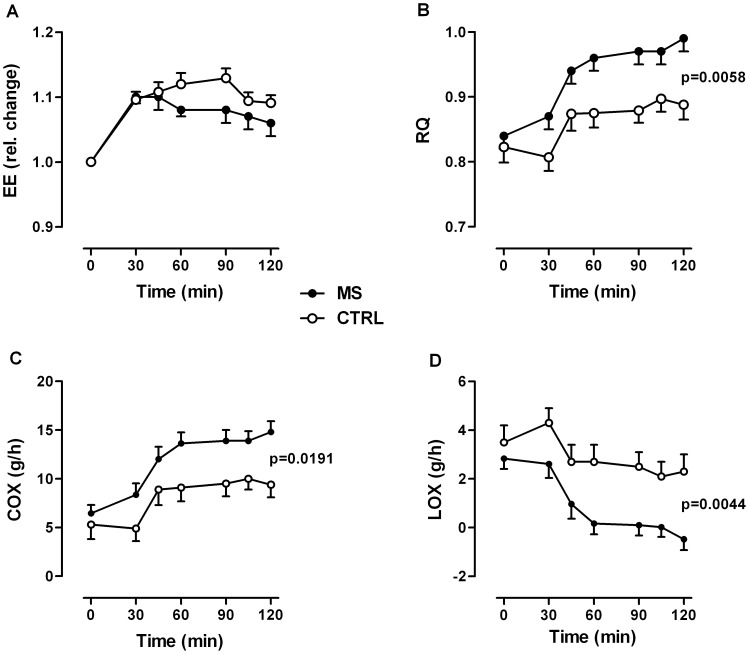
Systemic energy metabolism measured by indirect calorimetry (canopy device). (A) Energy expenditure (EE, relative changes), (B) respiratory quotient (RQ), (C) carbohydrate oxidation (COX) and (D) lipid oxidation (LOX) in MS patients (n = 16) and control subjects (n = 14) before and after an oral glucose load. The elevated RQ value in MS patients indicates increased carbohydrate oxidation. Data are given as means ± SEM. Group difference *P* value in graph, group*time interaction effects for RQ *P* = 0.0008, COX *P* = 0.0016, LOX *P* = 0.0009, all by Brunner analysis.

EE increased similarly within the first 30 min after glucose. Then, EE rather continued to increase within the next 30 min in controls, whereas it started to decrease in patients. However, at no time point postprandial EE was significantly different in patients vs. controls (Fig. 3A). Fasting RQ was almost identical between the groups. In controls, RQ decreased slightly within the first 30 min after glucose, followed by an increase within the following 15 and reaching a maximum 45 min after glucose. Then, RQ remained at that maximum level until the end of testing (Fig. 3B). In patients, however, RQ increased slightly but immediately within the first 30 min after glucose, followed by a stronger increase within the next 15 min and reaching a maximum 60 min after glucose. Then, RQ remained also at that maximum level until the end of testing (Fig. 3B). At all postprandial time points, RQ values were significantly higher in patients vs. controls, indicating higher postprandial carbohydrate oxidation rates (Fig. 3C) and, therefore, lower lipid oxidation rates (Fig. 3D).

**Figure 4:**
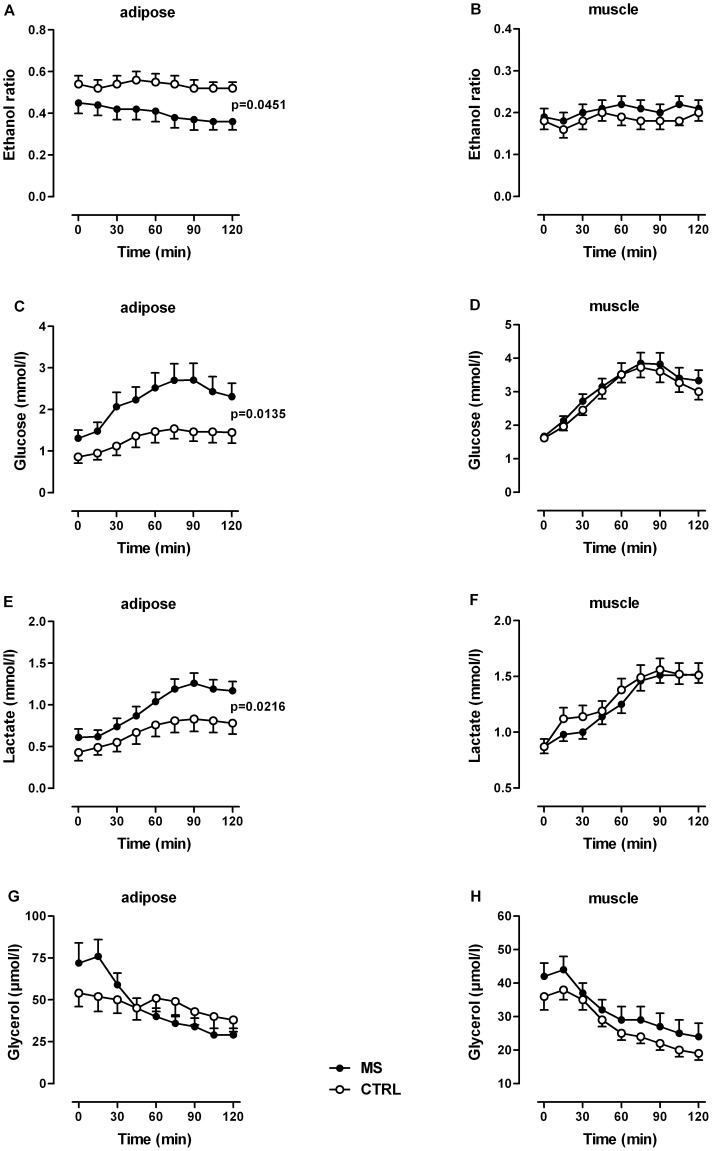
Adipose tissue and skeletal muscle microdialysis. (A, B) Ethanol ratio and dialysate concentrations of (C, D) glucose, (E, F) lactate, and (G, H) glycerol in adipose tissue and skeletal muscle in MS patients (adipose, n = 15; muscle, n = 16) and in control subjects (n = 16) before and after an oral glucose load. Data are given as means ± SEM. *P* values in graphs indicate group differences; group*time interaction effects were significant in adipose for lactate (*P* = 0.0225) and glycerol (*P* = 0.0001), all by Brunner analysis.

In adipose tissue, baseline ethanol ratio was significantly lower and therefore tissue perfusion significantly higher in patients vs. controls (Fig. 4A). After glucose, the ethanol ratio remained rather unchanged in controls whereas it decreased slightly but not significantly in patients. At all postprandial time points, ethanol ratio was significantly higher in patients vs. controls (Fig. 4A). Fasting dialysate glucose levels were higher and the postprandial increase was significantly greater in patients vs. controls, obviously a consequence of the about 2-fold greater tissue perfusion in patients vs. controls (Fig. 4C). Parallel to the greater glucose supply, baseline and postprandial dialysate lactate levels were also significantly higher in patients vs. controls (Fig. 4E). Taking into account the greater tissue perfusion in patients vs. controls, which leads not only to an increased substrate (glucose) supply but also product (lactate) removal, adipose tissue lactate production should be even higher than indicated by the dialysate levels. [28] In patients, glucose seems to be converted preferentially to lactate. In adipose tissue, glucose can be converted either to glycerol-3-phosphate for triacylglycerol synthesis in a liponeogenic state or to lactate in a rather lipolytic or insulin-resistant state. Baseline dialysate glycerol levels were higher in patients vs. controls (Fig. 4G). After glucose administration, dialysate glycerol levels decreased in both groups, in patients to a greater extent than in controls, reaching comparable values at the end of testing (Fig. 4G). Again, because of the greater tissue perfusion in patients vs. controls, glycerol production should be much higher than indicated by the dialysate glycerol levels, indicating a higher lipolytic state in patients vs. controls.

In skeletal muscle, baseline and postprandial ethanol ratio did not differ significantly between the groups (Fig. 4B). Within both groups, ethanol ratio did not change significantly throughout the test. Thus, tissue perfusion had no influence on postprandial changes in dialysate marker metabolite levels. Fasting dialysate marker metabolite levels did not differ significantly between the groups, and after glucose, dialysate glucose (Fig. 4D), lactate (Fig. 4F) and pyruvate (data not shown) levels increased and glycerol levels (Fig. 4H) decreased in a normal way without any significant differences between the groups. For both groups, baseline pyruvate concentrations were about 20 *µ*mol/L, and increased to 70 *µ*mol/L within 120 min after glucose. Although not significant, both fasting and postprandial dialysate glycerol levels were slightly higher in patients vs. controls (Fig. 4H).

**Figure 5:**
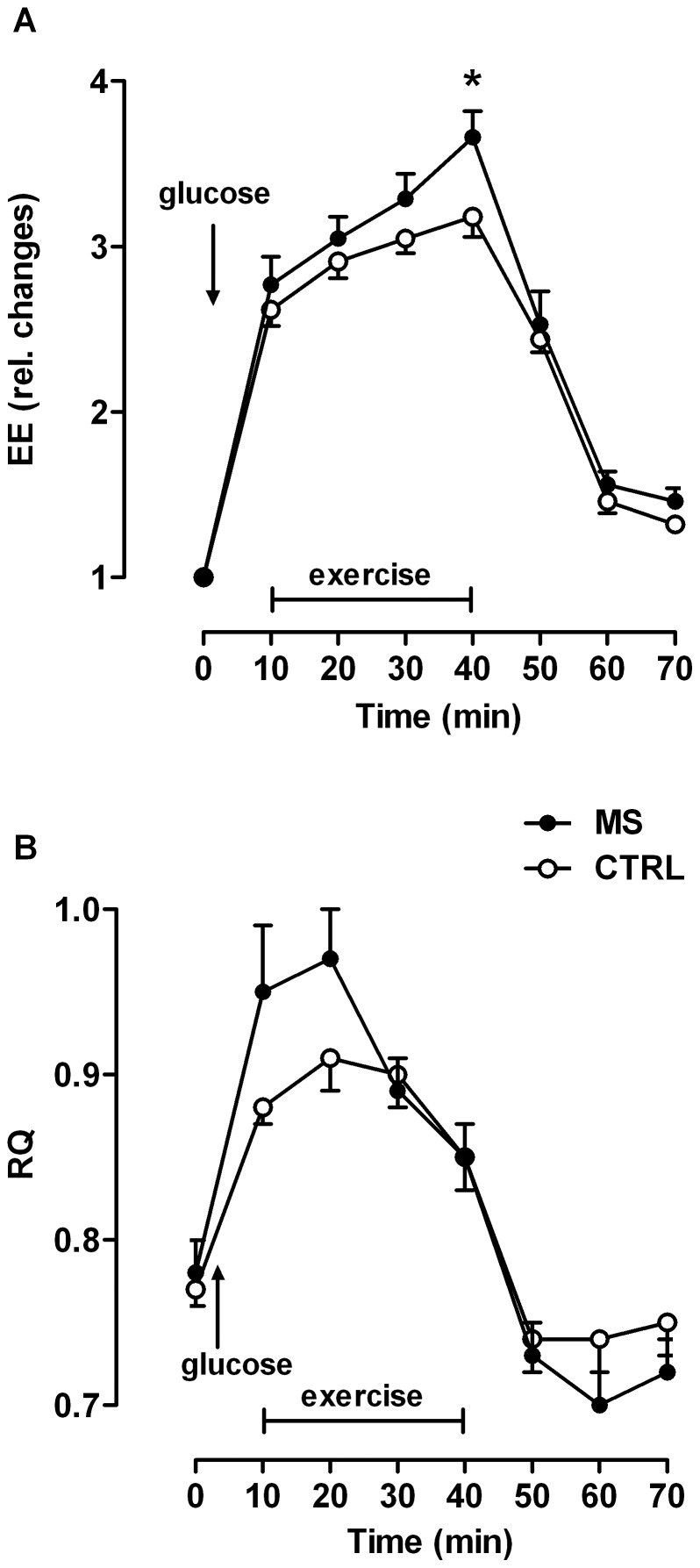
Systemic energy metabolism measured by indirect calorimetry (metabolic chamber). (A) Energy expenditure (EE) and (B) respiratory quotient (RQ) in MS patients (n = 12) and control subjects (n = 12) at rest and during bicycle exercise after an oral glucose load (OGL). EE after 40 min of exercise *P* = 0.0243, by Mann-Whitney-U test.

After beginning exercise, EE increased immediately within the first 10 min in both groups. EE reached a steady state within the next 20 min in controls. In contrast, EE continued to increase in MS patients, although more slowly and was significantly higher after 40 min of moderate exercise, compared to controls (Fig. 5A). During the recovery phase, EE returned to baseline levels in both groups (Fig. 5A). Resting RQ was again slightly higher in patients vs. controls, indicating a higher carbohydrate and lower lipid oxidation rate (Fig. 5B). During exercise, RQ and therefore carbohydrate oxidation, increased more strongly and to higher values in patients vs. controls during the first 20 min (Fig. 5B). Whereas the RQ was rather stable in controls between 20 and 30 min of exercise, it started to decrease strongly and immediately in patients after 20 min of exercise. During the recovery phase, RQ values decreased in both groups below baseline values and this to a greater extent in patients vs. controls (Fig. 5B).

## Discussion

We tested the hypothesis that metabolic flexibility is reduced in MS patients. We reasoned that such a state-of-affairs could contribute to their poor muscle strength, decreased performance, and exercise intolerance. We studied the metabolic response after glucose in fasted MS patients and healthy control subjects either at rest or during exercise. We found that at rest, fasting and postprandial carbohydrate oxidation was greater in MS patients compared to controls, while lipid oxidation was reduced. The postprandial EE was similar between the groups. The lipolytic activity in adipose tissue both before and after glucose was increased in MS patients compared to controls, but not in muscle. During exercise, MS patients exhibited a stronger initial increase in carbohydrate oxidation compared to controls. This response was followed by an immediate and rapid increase in lipid oxidation, without an intermediate metabolic steady state. We suggest that these abnormal responses could contribute to reduced physical performance.

A well-balanced interaction between sensory and somatic neurons is a prerequisite for coordinating and adjusting the different functions of the human body. At the somatic site, motor neurons control muscle contraction and visceral neurons the metabolic and hemodynamic adaptation during any physical activity. In MS patients, sensory and somatic neurons are irreversibly damaged leading to the diverse appearance of the disease. [29] However, muscle weakness, early fatigue and exercise intolerance are key symptoms in MS patients. During exercise, the nervous system affects metabolism directly through stimulation of lipolysis and indirectly through stimulation of muscle contraction, which in turn is linked to various metabolic adjustments. [30] Additionally, an impaired function of the nervous system could differently affect systemic and local tissue metabolism. To date, it is not clear whether skeletal muscle weakness and fatigue are rather consequences of denervation and disuse or of any other intrinsic mechanism.[31]–[33].

Interestingly, our patients presented a slightly higher carbohydrate oxidation rate than controls after a 12 h overnight fast. This result could be a consequence of greater glycogen stores or a reduced resting metabolic rate with impaired lipid mobilization and oxidation. However, resting metabolic rate was comparable between the groups. Shortly after glucose, healthy controls responded with a decrease in RQ and, therefore, increase in lipid oxidation as a consequence of an increased sympathetic activity due to gastric distension. [34] Then, RQ increased as expected, due to an increase in carbohydrate oxidation. However, we did not observe an initial RQ decrease in patients. Instead, the RQ began to increase immediately after glucose and finally reached much higher values when compared to controls. Postprandial (diet-induced) thermogenesis is also mediated partly by sympathetic activation. Although not significant, postprandial thermogenic response was slightly blunted in our patients. Both, a higher carbohydrate oxidation rate and a blunted thermogenic response after glucose could result from autonomic dysfunction. [35], [36] Autonomic dysfunction is a common phenomenon in MS patients. Particularly affected are bladder, bowel, cardiovascular function, sleep, sexual and sweat organs. [37] Accordingly, overall metabolic regulation could also be affected in MS patients.

Postprandial RQ values were not only higher at rest but also during a subsequent bicycle exercise over 40 min at a moderate intensity in patients vs. controls, although the difference was not significant. Interestingly, the increase in RQ was also immediate in patients vs. controls after starting bicycling, followed by a prompt decrease after reaching the maximum, even below resting values during the recovery period. During exercise, normally a mixture of carbohydrates/lipids is used for fuelling the increased energy production. The higher the intensity the greater is the carbohydrates-to-lipids ratio used as substrate for energy production. According to the glucose-fatty acid cycle by Randle et al. [38], fatty acids reduce glucose oxidation and vice versa. At low intensity exercise, normally lipids contribute significantly to energy production. Lipid mobilization and oxidation are regulated by the sympathetic nervous system. Our data indicate a delayed sympathetic activation and lipid mobilization. However, at least at the end of exercise, energy expenditure was higher in patients vs. controls indicating a reduced degree of effectiveness of muscle work in patients even at low but standardized work load (0.5 W/kg body weight). This difference might increase further with higher exercise duration or intensity.

The so far discussed abnormalities in MS patient´s energy metabolism do not indicate an altered metabolic flexibility due to mitochondrial dysfunction. In primary cultured myotubes established from healthy lean and obese type 2 diabetics, the variation in intramyocellular lipid oxidation *in vitro* was inversely related with fasting RQ *in vivo* of all subjects. [39] Also, within a stable energetic background, an increased mitochondrial mass in human myotubes was not directly correlated to an increased substrate oxidation as expected from skeletal muscle *in vivo* but, surprisingly, with a reduced complete lipid oxidation. Furthermore, physical inactivity decreases mitochondrial content and oxidative capacity of skeletal muscles *in vivo*. [40] However, in our study, ambulatory patients were included with a median EDSS of 2.0 and normal to moderately impaired mobility. Sedentary controls were closely matched for physical activity level. Since activity during work and leisure did not differ between the groups our findings cannot entirely be explained by reduced physical activity in the MS patients. Also, lean body mass of our patients was slightly lower (−5%) than in controls which is in line with another study. [41] The immediate increase in RQ, and therefore carbohydrate oxidation after the glucose load at rest and during exercise argues against impaired metabolic flexibility due to mitochondrial dysfunction.

The results regarding the systemic metabolic response to glucose were partly confirmed by our data regarding the local hemodynamic and metabolic response of adipose tissue and skeletal muscle to glucose while resting. In skeletal muscle, dialysate glucose increased about 2.5-fold, dialysate lactate about 2-fold, and dialysate pyruvate about 3-fold after glucose. Baseline and postprandial tissue perfusion did not differ between the groups. Hence, skeletal muscle oxidative glucose metabolism should be unaffected in MS patients as incited by our calorimetric observations. This finding implies also a proper function of the respiratory chain, especially of complex I, which is very important for carbohydrate oxidation. Defects in complex I of the mitochondrial respiratory chain as reported in Huntingtońs disease seem to be rather unlikely in MS patients at least at a median EDSS score of 2.0. [7], [8] However, we looked just at the metabolic response after glucose either at rest or during exercise. The picture might differ when studying muscle metabolism under fasting conditions during exercise. In adipose tissue, we observed a rather lipolytic state, indicated by higher baseline and postprandial dialysate glycerol levels and also higher postprandial dialysate lactate levels. This might indicate a local insulin resistance. In a recent study on obese patients we found that systemic monocyte activation is associated with tissue-specific changes in glucose and lipid metabolism which may be explained in part by monocyte/macrophage infiltration of adipose tissue which appears to interfere with local insulin responsiveness. [17] Systemic and local monocyte activation is a common feature of both MS and obese/diabetic patients. [42] The chronic inflammatory process in MS patients might affect specifically adipose tissue metabolism. However, we have not tested for that. Instead, is has to be mentioned, that all our patients were on glatiramer (copolymer 1, copaxone®) for at least 12 months when studied. Glatiramer is injected daily into adipose tissue. It has been reported that prolonged treatment with glatiramer leads to localized lipoatrophy. [43] Obviously, glatiramer can activate lipolysis, specifically in adipose tissue.

The strength of our study is that we studied MS patients and healthy controls closely matched for gender, age, body composition and physical activity level. Furthermore, MS patients were comparable with respect to EDSS score and medication. However, duration of the disease might differ within patients due to a variable time interval between first outbreak and diagnosis of the disease. Despite good matching, the sample number of patients was rather small. Furthermore, systemic and local markers for inflammation were not determined. Also, we did not test specifically for autonomic nervous system function. In future studies, MS patients should also be studied during exercise after overnight fasting, but without glucose and during graded (0.5 and 0.75 W/kg body weight) exercise intensities to characterize more precisely lipid mobilization and oxidation and autonomic dysfunction during exercise.

In summary, MS patients responded with a more highly increased carbohydrate oxidation rate after an oral glucose load than healthy controls, both at rest and during exercise at a moderate work load. At rest, adipose tissue was characterized by a higher lipolytic activity before and after the glucose load, which might be caused by either local monocyte/macrophage infiltration due to systemic inflammation or glatiramer injections into adipose tissue. Skeletal muscle did not show any signs of mitochondrial dysfunction.

In conclusion, glucose tolerance is not impaired in MS patients. At rest, there is no indication for metabolic inflexibility or mitochondrial dysfunction in skeletal muscle. The increased adipose tissue lipolytic activity might result from glatiramer treatment. Autonomic dysfunction might cause dysregulation of postprandial thermogenesis at rest and of lipid mobilization during exercise.
